# Association study of novel single nucleotide polymorphisms of androgen receptor and estrogen receptor-α genes with male infertility in Northwest of Iran: A case-control study

**DOI:** 10.18502/ijrm.v20i6.11446

**Published:** 2022-07-06

**Authors:** Elham Ghadirkhomi, Seyed Abdolhamid Angaji, Maryam Khosravi, Mohammad Reza Mashayekhi

**Affiliations:** ^1^Department of Genetics, Faculty of Biological Science, North Tehran Branch, Islamic Azad University, Tehran, Iran.; ^2^Department of Cell and Molecular Biology, Faculty of Biological Sciences, Kharazmi University, Tehran, Iran.; ^3^Department of Biology, Faculty of Biological Sciences, North Tehran Branch, Islamic Azad University, Tehran, Iran.; ^4^Department of Genetics, Faculty of Biological Science, Tabriz Branch, Islamic Azad University, Tabriz, Iran.

## Abstract

**Background: **Observational evidence on the association of novel single nucleotide polymorphisms (SNPs) of androgen receptor *(AR)* and estrogen receptor-α *(ER- α )* genes with odds of male infertility are rare. This is particularly relevant in the Iranian population where male infertility is relatively high. **Objective: **This study was designed to investigate the relationship between different SNPs of these genes and male infertility among the Iranian population. **Materials and Methods:** The present project was a population-based, case-control study conducted on 120 idiopathic azoospermia or severe oligospermia infertile cases alongside 120 age-matched subjects enrolled as controls. Overall, 3 variants from the *AR* gene and 2 variants from *ER- α* were genotyped - *ARrs137852568*, *ARrs137852599* and *AR rs137852563,* and* ER- α rs796065354* and *ER- α rs104893956- *using amplification refractory mutation system methods. **Results: **The obtained results indicated a significant association between *AR rs1378525568* TT genotype as well as *AR rs137852599* C allele with odds of male infertility (OR: 0.433, CI: 0.197-0.951 and OR: 0.545, CI: 0.304-0.978, respectively). Other variants of *AR* were not related to male infertility. A significant association was noted between predisposition polymorphism *ER- α rs796065354* genotypes with male infertility. This significant association was not seen between* ER- α rs104893956* and the risk of idiopathic azoospermia or severe oligospermia. Heterozygote overdominance was also observed in *ESR rs796065354* but not in the other variants studied. **Conclusion: **Pieces of evidence were found on the association of novel polymorphisms of *AR *and *ER- α* with male infertility among the Iranian population. However, larger studies are warranted to confirm our findings.**Key words:** *Male infertility, Androgen receptor, Estrogen receptor- α .*

## 1. Introduction

Infertility is a universally common issue defined as a failure to achieve pregnancy after a year of unprotected intercourse (1). According to the World Health Organization report, around 60-80 million couples of reproductive age suffer from infertility worldwide (2).

Although a complex network of environmental factors and genetic aberrations play a crucial role in the etiology of male infertility, the main cause of impaired spermatogenesis is unknown (3). In the case of spermatogenesis, androgen receptor (*AR*) mediates the androgen signaling in the testis. It has been pinpointed that disruption of the *AR*-signaling pathway results in a disturbance of maintenance of spermatogonial numbers, blood-testis barrier integrity, completion of meiosis, adhesion of spermatids, and spermiation (4, 5). Due to the fundamental effects of *AR* in spermatogenesis, various gene polymorphisms of *AR* concerning male infertility have been studied. Several meta-analyses have reported the association between *AR* gene CAG repeat length polymorphisms and male infertility (6-9). Despite these conclusions, some population-based primary studies did not find any significant associations between *AR* gene CAG as well as GGN repeat length polymorphisms and infertility among their male population (8, 10, 11).

However, a recent local survey in southwest Iran has revealed that GGN repeat length polymorphisms are one important polymorphism leading to male infertility among the Iranian population (12). These findings have led to the hypothesis that other polymorphisms in the* AR* gene should be examined which might be considered a risk factor for male infertility. Another hormonal receptor that was studied is estrogen receptor alpha (*ER- α *). Female hormones and their receptor gene polymorphisms, in particular estrogen receptors, have been challenged in elucidating their role in male infertility (13). It should be borne in mind that similar to *AR, ERs* also contribute to several steps of spermatogenesis such as modulated sperm metabolism, reduced testicular size, and severe oligospermia. Besides, gene expression of *ER* is significantly reduced in Sertoli cell-only tests (14). Evidence has added new significant findings on the association between *ER* gene polymorphisms, especially p and x alleles, and increased male infertility (10, 15).

Most of the previous studies have focused on some limited *ER* gene polymorphisms and additional information is needed on the association between *ER* polymorphisms and male infertility. Considering the high infertility rate among men, especially Iranian men, understanding the main genetic factors, in particular sex hormone-related contributors, might shed light on the cause and mechanisms of male infertility which can be used in therapeutic strategies. We are aware of the lack of data on various hormonal gene polymorphisms and the risk of male infertility. Therefore, it seems that other observational studies are needed to elucidate novel gene polymorphism of *AR* and *ER- α* and their causative role in the risk of infertility among men.

The present case-control study, therefore, examined the association of different polymorphisms of *AR* and *ER- α* genes with male infertility among the Iranian population.

## 2. Materials and Methods

### Participants 

This population-based, case-control study was carried out in 120 infertile men aged 25-45 yr presenting at the infertility treatment center of Valiasr hospital, Tabriz, Iran from March 2018 to July 2019. All cases were of idiopathic azoospermia or severe oligospermia (sperm count < 5 × 10^6^/ml) and their azoospermia was diagnosed and confirmed by a specialist. Participants with any identified causes were excluded from the study. All infertile cases were assessed by physical examination and all necessary hormonal and genetic tests including karyotyping, micro-deletion of the Y chromosome, and cystic fibrosis transmembrane conductance regulator mutations. The controls were 25-45 yr old and included 120 healthy men who had normal semen tests and at least 1 child at the time of the study. To determine the quality and quantity of sperm samples, semen analysis was done twice for all participants using microscopic examination methods according to the World Health Organization standard values (16).

### Extraction of peripheral blood DNA 

Blood samples were collected in an ethylenediaminetetraacetic acid - containing vacutainer tube (Greiner Bio-One, Germany) from the 240 randomly chosen individuals. Samples were stored at -80ºC and the genomic DNA was extracted from all participants' blood samples using a rapid extract polymerase chain reaction (PCR) kit (PCR Biosystems Company, UK) according to the manufacturer's protocol. The concentration and quality of extracted DNA were measured at 260 nm and 280 nm (A260/280) using a Nano Drop 2000 spectrophotometer (Ds-11 spectrophotometer, Denovix company, USA).

### Genotyping

The allele-specific PCR or amplification refractory mutation system method was applied to identify the genetic polymorphisms. Genotype analysis for the *ER- α* gene was done according to the tetra-primer amplification refractory mutation system PCR method. PCR primer sets were designed and optimized using primer 3 version 4.1.0 and primer 1 software. The quality and specificity of primers were analyzed by Oligo Analyzer software and primer blast on the NCBI website. To amplify the out target genes, a ready-to-use mastermix was used with a volume of 13 μL (PCR Biosystems Company, UK). The PCR reactions were performed with a final volume of 25 μL. PCR products were analyzed by 2% gel electrophoresis using novel juice stain (Cat.No.LD001-1000, GeneDireX, Taiwan). Samples were re-genotyped to confirm the results.

### *AR* single nucleotide polymorphisms (SNPs)

The *AR*A > T (rs137852568) was amplified by using a common reverse primer (5'-ATC TGAAAGGGGGCATGAGC- 3'), wild type forward primer (5'-GTTCACTTTTGACCTGCTAAT AA-3'), and mutant type forward primer (5'-GTT CAC TTT TGA CCT GCT AAAT-3'). The cycling condition for wild-type amplification was 95 o C for 3 min for the first cycle, 95 o C for 30 sec, 57 o C for 30 sec, 72 o C for 1 min for 35 cycles, and the final extensional time of 7 min at 72 o C. All cycling conditions for mutant and wild types were the same. The primers used to amplify *AR* A > C (*rs137852599*) were as follows: common forward primer 5'-AGT CTC TCTTCCTTCCCA ATA G-3', wild type reverse primer 5'-TCAGGCTGGTTGTTGTCG T-3', and mutant type reverse primer 5'-TCAGTCTGGTTGTTGTCG G-3'. The cycling condition for all primers was 95 o C for 3 min for the first cycle, 95 o C for 30 sec, 55 o C for 30 sec, 72 o C for 30 sec for 30 cycles, and the final extension in 72 o C for 5 min. Primers for AR G > A (*rs137852563*) were as follows: common reverse primer 5'-GGG CAG AAA AGC ACC AGA CA-3', wild type forward primer 5'-GCT TGT ACA CGT GGT CAA GTT G-3', and mutant forward primer 5'-GCT TGT ACA CGT GGT CAA GTT A-3'. PCR conditions for both wild and mutant types were 95 o C for 3 min at the first cycle, 95 o C for 45 sec, 58 o C for 30 sec, and 72 o C for 1 min for 30 cycles. The final extension was at 72 o C for 5 min. The sizes of the PCR products were 185, 224, and 132 base pairs (bp) for the rs137852568, *rs137852599*, and *rs137852563* polymorphisms, respectively.

### *ER*-α SNPs

To amplify the *ER- α* A > G (*rs796065354*), 4 primers were used as follows: forward inner primer 5'-CTG ACCAAACGCTCTAAG AA-3', reverse inner primer 5'-ACAAAGCCAGGCTGT TCC-3', forward outer primer 5'-GCAGGGATACGAAAAGAC C-3', and reverse outer primer 5'-CTTACC TGGCACCCTCTT C-3'. The cycling condition for all primers was in this manner: for the first cycle, 95 o C for 5 min, then 95 o C for 40 sec, 55 o C for 35 sec, 72 o C for 45 sec, for 29 cycles, and final extension at 72 o C for 7 min for all types of primers. The other polymorphism of *ER- α* C > T (*rs104893956*) was amplified using forward inner primer 5'-CCAGGCAAACTTCAGATAATC- 3', reverse inner primer 5'-TTCTTACACCCTGGCGTC A-3', forward outer primer 5'-ACA CAA TTT CCC CTC AAG G- 3', and reverse outer primer 5'-TCTCTTAGGATCTGCTCATAG G-3' with the cycling condition 95 o C for 5 min for the first cycle, 95 o C for 30 sec, 55 o C for 30 sec, 72 o C for 45 sec for 29 cycles, and the final extension time of 7 min at 72 o C. The cycling conditions for all primer types were the same. The *rs104893956* polymorphism PCR products were 443, 252, and 230 bp for forwarding outer and reverse outer, forward inner and reverse outer, and reverse inner and forward outer, respectively. The PCR products of the *rs104893956* polymorphism were as follows: 345, 213, and 169 bp for outer forward and outer reverse, inner forward and outer reverse, and outer forward and inner reverse, respectively.

### Ethical considerations

This study was conducted following the ethical principles, the national norms, relevant guidelines, and regulations for conducting medical research in Iran. Written informed consent was obtained from all eligible subjects. The project was approved by the Science and Research Branch of Tehran's Islamic Azad University's Ethical Committee, Tehran, Iran (Code: IR.IAU.SRB.REC.1398.001).

### Statistical analysis

The frequency of genotypes in the case and control groups were evaluated for Hardy-Weinberg equilibrium (HWE) and Chi-square was calculated. The association of the SNPs of *AR* and *ESR* with male infertility was assessed by using conditional logistic regression. Statistical analyses were carried out using SPSS version 22 (IBM, Armonk, NY, USA). P-values < 0.05 were considered significant.

## 3. Results

The concentration of the extracted DNA was in the range of 77-247 ng/μL. The obtained OD 260/280 was in the range of 1.78-1.89.

Table I shows the genotypic frequencies of *AR* and *ER- α* variants in the fertile and infertile men. A total of 3 variants for the *AR* gene and 2 variants for the* ER- α* gene were identified. There were no significant differences between the 2 groups in terms of *AR* *rs1378525568* and *rs137852563 *variants and *ER- α* gene *rs104893956*; however, there was a significant difference between cases and controls in terms of *ER- α* *rs796065354* (p = 0.001) and AR *rs137852599* (p = 0.040).

The associations of *AR* and *ER- α* polymorphisms with male infertility are shown in table II. We observed a significant association between *AR rs1378525568* TT genotype and odds of idiopathic azoospermiaor severe oligospermia compared with normal homozygote (p = 0.035, OR: 0.433, CI: 0.197-0.951). The HWE examination test revealed that this equilibrium for *AR rs1378525568* (A > T) was disrupted in healthy controls, not in cases (Chi^2^ = 6.0449 and 0.2721, respectively) and the additive pattern was considered. To examine whether HWE in a group was disrupted or not, the calculated Chi-square was compared to the statistics table Chi-square. If the calculated Chi-square was bigger than the statistics table Chi-square (3.8), the H0 hypothesis was rejected, which would mean HWE was disrupted in that group, and otherwise the H1 hypothesis was rejected, which would mean the group was in HWE. In terms of *AR rs137852599*, there was a significant association between the C allele (recessive allele) and odds of male infertility compared with the dominant allele (OR: 0.545, CI: 0.304-0.978). Men with this polymorphism were at a 45% decreased risk of infertility. The examination of HWE for *AR rs137852599 *(A > C) indicated that both controls and cases were in HWE (Chi^2^ = 1.384 and 1.9438, respectively). Therefore, statistical analysis was performed based on the multiplicative pattern. There were no significant associations between *AR rs137852563* genotypes and infertility risk among men compared to the normal homozygote. For *rs137852563* (G > A), the HWE was disrupted in the 2 study groups (Chi^2^ = 58.6723 and 37.474 for controls and cases, respectively); therefore the additive model was used for statistical analysis.

The results showed a significant association between the *ER- α rs796065354 *genotypes (AG/AA, GG, GA+GG, and GA/GG+A) and the risk of idiopathic azoospermiaor severe oligospermia compared to the normal homozygote. For *ER- α rs796065354* (A > G), none of the cases or controls were in HWE (Chi^2^ = 28.3334 and 13.0515, respectively), which showed the additive pattern. Heterozygote over dominance was also observed in this SNP. In terms of *ER- α rs104893956*, there was no significant relationship between any genotypes of CT/CC, TT, CT+TT, and CT/TT+CC and male infertility compared with the normal homozygote. The HWE examination test for *ER- α rs104893956* (C > T) revealed that this equilibrium was disrupted in the healthy controls, not in the cases (Chi^2^ = 6.9657 and 0.1936, respectively) and the additive pattern was considered.

**Table 1:** Distribution of the *AR *& *ER- α* genotypes in the cases and controls


		**Cases (n = 120-121)**	**Controls (n = 119-120)**	P-value $o^{*}$	**MAF (ALFA)**
**Genotype**					
	***AR 1378525568*** ** AA**	60 (50.0)	52 (43.3)	
	***AR 1378525568*** ** AT/AA**	48 (40.0)	44 (36.7)	
	***AR 1378525568*** ** TT **	12 (10.0)	24 (20.0)	0.093	C > T T = 0.0
**Genotype**					
	***AR 137852599*** ** A**	220 (51.9)	204 (48.1)			
	***AR 137852599*** ** C**	20 (37.0)	34 (63.0)	0.040	A > C No data
**Genotype**					
	***AR 137852563*** ** GG**	55 (45.8)	63 (52.5)	
	***AR 137852563*** ** GA/GG**	26 (21.6)	17 (14.1)	
	***AR 137852563*** ** AA**	39 (32.5)	39 (32.5)	0.298	G > A A = 0.000006/1
**Genotype**						
	***ESR 796065354*** ** AA**	18 (15.0)	41 (34.2)			
	***ESR 796065354*** ** AG/AA **	89 (74.1)	73 (60.8)	
	***ESR 796065354*** ** GG**	13 (10.8)	6 (5.0)	0.001	A > G No data
**Genotype**					
	***ESR 104893956*** ** CC**	95 (79.2)	99 (82.5)			
	***ESR 104893956*** ** CT/CC**	23 (19.2)	17 (14.2)	
	***ESR 104893956*** ** TT**	2 (1.7)	4 (3.3)	0.466	C > T T = 0.0
Data presented as n (%). *P-values were obtained from Chi-square test. AR: Androgen receptor, ESR: Estrogen receptor, MAF: Minor allele frequency, P-value < 0.05 considered significant. Obtained p-value showed significant difference between case and control groups in *AR rs137852599* and *ESR rs796065354*					

**Table 2:** Odds ratios for male infertility across different genotypes of *AR* and *ESR*


		**OR (95% CI)**	**P-value**
**Genotype (additive model)**			
	***AR 1378525568*** ** AA**	1.00	
	***AR 1378525568*** ** AT/AA**	0.945 (0.544-1.643)	0.842
	***AR 1378525568*** ** TT**	0.433 (0.197-0.951)	0.035
	***AR 1378525568*** ** AT+TT**	0.765 (0.460-1.271)	0.301
	***AR 1378525568*** ** AT/AA+TT**	1.152 (0.684-1.938)	0.595
**Genotype (multiplicative model)**			
	***AR 137852599*** ** A**	1.00	
	***AR 137852599*** ** C**	0.545 (0.304-0.978)	0.040
**Genotype (additive model)**			
	***AR 137852563*** ** GG**	1.00	
	***AR 137852563*** ** GA/GG**	1.752 (0.861-3.564)	0.120
	***AR 137852563*** ** AA**	1.145 (0.646-2.031)	0.642
	***AR 137852563*** ** GA+AA**	1.330 (0.800-2.211)	0.272
	***AR 137852563*** ** GA/AA+GG**	1.660 (0.847-3.251)	0.137
	***ESR 796065354*** ** AA**	1.00	
	***ESR 796065354*** ** AG/AA**	2.777 (1.472-5.239)	0.001
	***ESR 796065354*** ** GG**	4.935 (1.619-15.048)	0.003
	***ESR 796065354*** ** GA+GG**	2.941 (1.571-5.507)	0.001
	***ESR 796065354*** ** GA/GG+AA**	1.848 (1.067-3.201)	0.027
**Genotype (additive model)**			
	***ESR 104893956*** ** CC**	1.00	
	***ESR 104893956*** ** CT/CC**	1.410 (0.709-2.803)	0.326
	***ESR 104893956*** ** TT**	0.521 (0.093-2.911)	0.684
	***ESR 104893956*** ** CT+TT**	1.241 (0.651-2.364)	0.512
	***ESR 104893956*** ** CT/CC+TT**	1.437 (0.724-2.851)	0.299
*AR*: Androgen receptor, *ESR*: Estrogen receptor. Association of different genotypes of each SNP with male infertility was assessed using Chi-square test			

## 4. Discussion

Due to the fundamental effects of genetic contributors on the etiology of infertility, examining the SNPs is of high priority. Among the genes involving the fertilization process, in particular, in men, hormone-related genes and their receptors due to multifunctional properties in male physiology and spermatogenesis are strongly targeted in various infertility studies. *AR* as one of the interesting receptors has been studied frequently and its role in spermatogenesis, the survival of germ cells, and their maturation is well-defined (5, 17).

The results of this population-based, case-control study suggested that *AR rs1378525568* TT genotype and *AR rs137852599* C allele have a protective effect on fertility and may reduce idiopathic azoospermia or severe oligospermia risk. We also observed an association between* ER- α rs796065354* genotypes (AG/AA, GG, GA+GG, and GA/GG+A) and odds of infertility among men. However, the results failed to find any significant association between other polymorphisms of *AR* and *ER- α* genes with the risk of infertility among our study population. To the best of our knowledge, this study is among the first case-control studies examining the association between specific polymorphisms of *AR* and *ER- α* genes with the risk of male infertility among the Iranian population.

Most meta-analysis studies have concluded that the increase in CAG repeat length in *AR *is associated with male infertility (6-9). However, the findings of an up-to-date meta-analysis which were classified by region and sub-types of male infertility were inconsistent. It seems that Caucasian populations are more susceptible to this polymorphism due to *AR* dysfunctions (7-9). We found for the first time, an inverse association between* AR rs1378525568* TT genotype and *AR rs137852599* C allele with male infertility. Most previous studies have focused on CAG repeat length polymorphisms, and their results are in contrast to our findings (12, 18, 19). Besides, some studies failed to find any significant relationship between *AR* polymorphisms and the odds of various sub-types of male infertility (20). However, similar to other reports, we failed to find a significant association between other alleles of *AR rs1378525568* as well as *AR rs137852599* and *AR rs137852563 *variants, and the fertilization ability of men. It should be mentioned that previous data on *AR* polymorphisms have been limited to the CAG repeat length polymorphism, so previous findings differ from ours which has studied novel polymorphisms. Moreover, some basic differences concerning environmental factors, study population, and sample size might contribute to the conflicting findings between studies. Overall, it is suggested that further studies are required to investigate the relationship between different types of* AR* polymorphisms and male reproductive function.

Despite estrogens and their receptors (*ERs*; including *ER- α , ER- β *, and *ER- γ *) being conventionally regarded as female hormones, there is a wide range of investigation showing their profound effects on the male reproductive system (3). In the case of infertility, the exact physiological role of estrogens in spermatogenesis is not clearly understood. However, based on recent studies, they are regarded as a “survival factor”. According to this concept, the absence of* ER- α* results in reducing the epididymis sperm content, sperm motility, and fertilizing ability (21, 22). It seems that *ER- α* confers a stronger estrogen effect. We found, as the first case-control study, *ER- α rs796065354* genotypes (AG/AA, GG, GA+GG and GA/GG+A) might increase the odds of male infertility. Similar to *AR*, *ER- α* gene polymorphisms investigated in previous observational studies were different from the polymorphisms we studied. However, it has been reported that the (TA) $o_{n}$ repeat polymorphism may negatively influence spermatogenesis (22). Some studies have demonstrated that XbaI and PvuII polymorphisms of the *ER- α* gene are associated with the risk of male infertility (15, 23).* ER- α* gene polymorphisms might affect sperm quality in men. *ER- α* 397T/T and *ER- α* 351A/A genotypes were associated with lower sperm motility in men with oligozoospermia (24). Semen variables including sperm count, motility, velocity, and morphology as well as sperm acrosin activity were significantly higher among infertile oligoasthenoteratozoospermia men who had pp and xx genotypes (15). Nevertheless, we found no significant relationship between *ER- α rs104893956* genotype and idiopathic azoospermia or severe oligospermia among our study population. It should be noted that our studied polymorphisms were completely different from other studies; therefore, this may lead to some inconsistency. Also, some controversial findings might be a reflection of the difference in study design and population. Similar to *AR*, it is suggested to conduct more observational studies to elucidate the association between different types of *ER- α* genotypes with male infertility.

The exact mechanisms through which *AR* and *ER- α* polymorphisms influence male infertility risk are complex and not well-established. Given the role of *AR* and *ER- α* in spermatogenesis and male infertility, one might assume that binding of the *AR-*androgen complex to HSPs and the subsequent transfer of the complex to the nucleolus modify the transcription of various genes involved in spermatogenesis (5). Such classic genomic action was considered for *ER- α* where translocation of the *ER*- α -estrogen complex to the nucleolus, and its interaction with DNA-binding elements in the genome, alters the expression of steroid-responsive genes (25).

Being the first report on the association of novel polymorphisms of *AR *and *ER- α* with odds of male reproductive function, as well as the age-matched design that led to control of potential age-dependent confounders, are the strengths of the present study. However, some limitations should be considered in the interpretation of our findings. Control genotypes should be in HWE, as long as the population they are selected from is random and large in size. A significant result showing that controls are not in HWE could arise because of 1) random chance (one of every 20 markers tested will give a p-value < 0.05 by chance); 2) incorrect genotyping; and 3) heterogeneous population. Provided the controls are in HWE, the cases may then be tested. If the SNP has a true genetic effect that is not controlled by a multiplicative model, the cases will not be in HWE. The test has little power to detect small departures from HWE (26).

Environmental factors, smoking, high-risk jobs, residence in high-risk areas, etc. are some confounders that were not included in this study. Therefore, the generalizability of the findings should be done cautiously.

## 5. Conclusion

In conclusion, we found evidence of an association between novel polymorphisms of *AR *and *ER- α* and male infertility among the Iranian population. Based on the results, *AR rs1378525568 *TT genotype and *AR rs137852599* C allele may have a protective effect on male fertility. In contrast, there was a significant association between predisposition polymorphism *ESR- α rs796065354* and male infertility that may be considered as a biomarker for male infertility. However, further studies with different ethnic populations and larger samples are needed to validate the findings.

##  Acknowledgements

The authors are thankful to Dr. Danaei for her help in sampling and medical diagnosis, and to Ms. Beikzadeh for data analysis. This research received no specific grant from any funding agency in the public, commercial, or not-profit sectors.

##  Conflict of Interest

The authors declare that there is no conflict of interest.
